# Southern Surgical and Gynecological Association

**Published:** 1889-08

**Authors:** 


### THE SOUTHERN SURGICAL AND GYNECOLOGICAL ASSOCIATION.

---

There lies before us a copy of the first volume of Transactions of the Southern Surgical and Gynecological Association, which has just been issued and distributed to the members. As this Association is of recent birth, and perhaps more or less of a stranger to most of our readers, we deem the occasion not inopportune to say a few words in regard to its scope and work.

Its organization was effected somewhat more than a year ago, and its first regular meeting was held in Birmingham in December last. The meeting was largely attended and was successful to an extent which surprised even the most sanguine advocates of the new enterprise. Judging from the record which lies before us, it may be truly said that the Association literally sprung into being "Minerva-like, full armed."

Its membership includes, with very few exceptions, all the best names in the South in the special lines of work indicated in the title of the organization, and covers the whole territory from Virginia to Texas, every State being worthily represented. Such a list of members is ample guarantee of future success and activity.

The papers contained in the volume of Transactions cover a variety of subjects, and do credit to their authors. They compare favorably with the work of any special society in this country, and show conclusively that the organization which has produced them fills the proverbial "long felt want." The South has heretofore failed to secure the recognition which it deserved in the so-called "national" special societies, and the large amount of good work in surgery and gynecology, which has been accomplished in this section of the country, has not been published to the world for the lack of an organization which should stimulate reports of such work and bring the workers into that intimate association which is universally recognized as conducive to the best results. There is no lack of able surgeons in the South, as is proven by the contents of the volume before us; while the mention of such names as McDowell, Sims, Thomas, Emmet, Bozeman and Battey—all Southern men—shows that the true home of modern gynecology is south of Mason and Dixon's line. The reason that the South has not played a more prominent *role* in the literature of these branches of medical science is found in the lack of such organizations as the Southern Surgical and Gynecological Association.

In one respect the gynecological portion of this volume differs—happily, we think—from the reports of most similar societies during the past few years. Abdominal surgery finds here its true place as subordinate to the curative methods of treatment. The true province of gynecology lies in the restoration of the integrity of the contents of the female pelvis. The removal of an organ is a confession of inability to restore it to health, and is therefore a reproach to medical science. While removal of organs is often necessary in the present state of our knowledge, yet to exalt destructive procedures above measures whose object is to restore organs instead of to destroy them, is calculated to place such destructive surgery in an improper light, and to divert attention from the true aim and end of all medical practice. Abdominal surgery is not the crowning glory of gynecology, as it is sometimes represented to be. There yet remains a higher plane to be reached when medical science shall obviate the necessity of extirpating important parts by teaching us how to restore them to health. It is gratifying to observe that the tendency of the work done at the Birmingham meeting was in this direction.

A large portion of the success of the Association is due to the indefatigable industry and perseverance of its able Secretary, Dr. W. E. B. Davis, of Birmingham, Alabama. But for him the society would never have been born. We extend to him our hearty congratulations upon the gratifying results of his efforts, and assure him that the Association will remain a permanent monument to his energy and zeal.

The next meeting will be held in Nashville, in November next, under the presidency of the eminent Dr. Hunter McGuire, of Richmond, Virginia. A large attendance is already assured and many valuable papers have been promised from the best workers and writers in the South. The profession is looking for brilliant results from this young but vigorous organization, and the present indications are that such expectations are in no danger of being disappointed.

